# The role of literal meaning in figurative language comprehension: evidence from masked priming ERP

**DOI:** 10.3389/fnhum.2014.00583

**Published:** 2014-08-04

**Authors:** Hanna Weiland, Valentina Bambini, Petra B. Schumacher

**Affiliations:** ^1^Department of English and Linguistics, Johannes Gutenberg University MainzMainz, Germany; ^2^Center for Neurocognition and Theoretical Syntax, Institute for Advanced Study IUSS PaviaPavia, Italy; ^3^Institute of German Language and Literature I, University of CologneCologne, Germany

**Keywords:** metaphor, metonymy, literal meaning, masked priming, N400, late positivity, experimental pragmatics

## Abstract

The role of literal meaning during the construction of meaning that goes beyond pure literal composition was investigated by combining cross-modal masked priming and ERPs. This experimental design was chosen to compare two conflicting theoretical positions on this topic. The indirect access account claims that literal aspects are processed first, and additional meaning components are computed only if no satisfactory interpretation is reached. In contrast, the direct access approach argues that figurative aspects can be accessed immediately. We presented metaphors (*These lawyers are hyenas*, Experiment 1a and 1b) and producer-for-product metonymies (*The boy read Böll*, Experiment 2a and 2b) with and without a prime word that was semantically relevant to the literal meaning of the target word (*furry* and *talented*, respectively). In the presentation without priming, metaphors revealed a biphasic N400-Late Positivity pattern, while metonymies showed an N400 only. We interpret the findings within a two-phase language architecture where contextual expectations guide initial access (N400) and precede pragmatic adjustment resulting in reconceptualization (Late Positivity). With masked priming, the N400-difference was reduced for metaphors and vanished for metonymies. This speaks against the direct access view that predicts a facilitating effect for the literal condition only and hence would predict the N400-difference to increase. The results are more consistent with indirect access accounts that argue for facilitation effects for both conditions and consequently for consistent or even smaller N400-amplitude differences. This combined masked priming ERP paradigm therefore yields new insights into the role of literal meaning in the online composition of figurative language.

## Introduction

Human communication often requires the construction of meaning that goes beyond the pure compositional computation of the literal meaning of the single sentence components. In contrast to popular believe, these non-literal utterances are not rare individual cases but an ever-present phenomenon in our daily communication (cf. Lakoff and Johnson, 1980). Several types of non-literal language have already been theoretically discussed and empirically investigated in the domain of experimental pragmatics and neuropragmatics (Noveck and Reboul, 2008; Bambini and Bara, 2012; Schumacher, 2013), however there are still some important remaining questions. In the following, we will first concentrate on the processing of metaphors since they play a prominent role in the theoretical discussion (cf. Grice, 1975; Sperber and Wilson, 1985; Gibbs, 1994; Giora, 1997; Glucksberg et al., 2001; Carston, 2010a). We will discuss the general underlying mechanisms in metaphor comprehension, then we will investigate the role of literal meaning aspects on early processing through a novel experimental design. This will be complemented by establishing a connection to one other type of non-literal language, namely metonymy. We report two experiments that investigated (i) the cognitive basis of metaphor and metonymy comprehension in German through event-related potentials (ERPs) and (ii) the role of literal meaning in figurative language processing by using the cross-modal masked priming technique in sentential context in combination with ERPs.

### Theoretical debate over figurative meaning

The contribution of literal meaning aspects^1^ during figurative processing marks one of the dividing lines between competing theories. While some theories suggest that the processor always starts from the literal meaning (indirect access account), others assume that only the relevant meaning is processed (e.g., direct access account, Relevancy Theory). In the following we will concentrate on these two extreme positions, since the relatively old debate between the indirect and direct access account still continues and hasn't been fully answered. In particular the question of the early contribution of literal meaning aspects that rests at the core of these two opposing views has not been settled yet. Of course, a range of theoretical approaches exist beside these poles that have adopted less extreme positions: Gibbs (2002) and Recanati (1995) for instance argue for literal meaning to play only a local role: it is activated for single words within a figurative utterance but the processing of the literal meaning of the whole figurative utterance is not required. Others consider literal meaning to linger in the background (cf. Carston, 2010b, but also Giora, 2008). Literal meaning is also suggested to be the important foundation for blending (cf. Fauconnier and Turner, 2002; Coulson and Oakley, 2005), respectively mapping processes (cf. Coulson and Matlock, 2001; Croft, 2002), merging of features (cf. Kintsch, 2000) or the activation of secondary cognitive representations (cf. Evans, 2010). Gentner and Wolff (1997) and Wolff and Gentner (2011) relate the role of literal meaning to the progress of the career of the metaphor. At the beginning, the metaphorical meaning is created via structural alignment of the components of the literal meaning, but in the course of repeated usage, the metaphorical meaning is stored in the lexicon (yielding a dead metaphor). Furthermore, non-literal language use encompasses many different phenomena, including irony, humor, hyperbole, simile, and so forth (cf. e.g., Giora, 1995; Carston, 2002; Sperber and Wilson, 2008; Gibbs and Colston, 2012). It is thus important to identify the differences and commonalities between the various types of figurative language comprehension. In the following, we focus on the link between metaphor and metonymy, which has not been investigated systematically yet (but see Gibbs, 1990; Rundblad and Annaz, 2010; Bambini et al., 2013 for initial developmental and behavioral findings).

In non-literal language processing^2^, the meaning of an utterance must be extended beyond the standard connotation. Not only the range of required modifications varies but also the range of possible interpretations. Metaphor (e.g., *These dancers are butterflies*) is the linguistic phenomenon that allows the greatest width of possible interpretations. Even in the simple form “X is Y,” one can imagine a reading in which the dancers are colorful, fluttering, light-footed, and so forth. Since ancient times, the understanding of metaphors has often been defined as transferring properties of a word or phrase, the source (e.g., *butterflies*), to an event, person or object, the target (e.g., *dancers*), where source and target are not directly connected (cf. the “transfer” Aristotle discussed in *Rhetoric*; see also Black, 1962; Lakoff, 1993).

How does a metaphoric reading emerge? This question has sparked a lot of debate (see e.g., Gibbs and Colston, 2012 for an overview). As mentioned above, we will concentrate on two extreme positions, the indirect and direct access view. The indirect access view (also labeled standard model) originates from the approaches by Grice and Searle. Grice (1975) assumed that a metaphor violates the conversational maxim of quality, but the addressee assumes the violation to be intentional and then seeks a meaningful interpretation by means of pragmatically driven implicature. Searle (1979) suggested that metaphors are processed in three steps. First the utterance is identified as not being literally interpretable, i.e., what is said is not what is meant. Second the addressee has to look for possible alternative interpretations of the utterance by comparison of properties. In the last step, the identified properties are checked for their sensicality. Accordingly, the possible interpretations of the metaphor are always achieved by going through the literal meaning. In terms of language processing, this approach would predict differences between literal and non-literal utterances, where any utterance is claimed to be first interpreted literally. These assumptions of the indirect access view have been criticized in subsequent work (cf. e.g., Sperber and Wilson, 1986; Giora, 1997; Gibbs, 2002).

The direct access view argues against the idea that the literal meaning is always accessed first (cf. Gibbs, 1994; Glucksberg, 2008). Originating from the idea that the understanding of metaphor is based on dual reference, Glucksberg (2008) for instance suggested that the processing of metaphor does not include more steps than the interpretation of literal utterances. Assuming that the vehicle (source) has a literal and a metaphorical reference, the processor only has to choose the appropriate one. For the metaphor *This lawyer is a shark*, the processor activates the metaphorical reference *shark* of the predator category that includes all properties relevant for the metaphor (e.g., aggressive, predatory, etc.) but none of the properties irrelevant for the metaphor (e.g., having fins). In a literal context (*This animal is a shark*), the literal reference, including properties like swimming, having fins, and so forth, is selected (see also Kintsch, 2000 for a computational account utilizing latent semantic analysis). Accordingly, literal and figurative meaning should be processed equally fast. These accounts further predict that the pre-activation of the literal meaning of the vehicle (e.g., *shark*) should hamper the processing of the metaphor (cf. Glucksberg, 2008:68: “[l]iteral meanings do not have unconditional priority, and so they are not necessarily easier to compute than nonliteral meanings.”).

A similar view has been advanced by Relevance Theory (Carston, 2002; Sperber and Wilson, 2008), where the linguistic content of any type of utterance (metaphoric, hyperbolic, literal, etc.) is underdetermined and the underlying processes should thus be the same. Utterance interpretation is guided by the Principle of Relevance and based on inferential reasoning. Two processes, narrowing and broadening, are involved in the construction of meaning, through which the addressee creates an ad hoc concept, including the relevant meaning range for the current context (cf. Carston, 2002, 2010a; Wilson and Carston, 2007). Although, the processing of literal meaning only involves the selection of the lexical meaning and no narrowing or broadening processes, the underlying inferential steps are suggested to be nearly identical in both cases (Sperber and Wilson, 1986). Hence, access to literal meaning is not obligatory. A newer relevance-theoretic approach by Carston (2010b) contemplated additional effort for the interpretation of figurative language. Based on empirical findings from Rubio Fernández (2007), Carston argued for the lingering of literal meaning even in figurative language processing. Accordingly, (extended) metaphors are appreciated and reflected upon with literal meaning aspects in mind. In terms of processing, this suggests that literal meaning aspects are accessible early on and active throughout metaphor processing.

### Experimental evidence on the role of the literal meaning

Previous experimental research indicates that costs are exerted during metaphor processing (for behavioral findings see e.g., Cacciari and Glucksberg, 1994; Noveck et al., 2001), which is further modulated by numerous factors like familiarity, appropriateness, context (for evidence at the behavioral and neural level see e.g., Gibbs, 1994; Giora, 1997; Bambini et al., 2011; Forgács et al., 2012). As far as ERP studies are concerned, several experiments have been conducted in different languages, e.g., in English (Coulson and van Petten, 2002, 2007; Lai et al., 2009; De Grauwe et al., 2010) French (Pynte et al., 1996), Hebrew (Arzouan et al., 2007) and Italian (Resta, 2012). All studies reported a more pronounced N400 for metaphors in contrast to literal control conditions. Hence, the N400 can be considered a stable component in the ERP-research on metaphor that is found for the processing of literary (Resta, 2012) and every-day metaphors, both verbal (Lai et al., 2009) and nominal (e.g., Pynte et al., 1996). Pynte et al. (1996) and Lai et al. (2009) also manipulated the conventionality of usage and the surrounding context. They reported a more pronounced N400 for all metaphors, with amplitudinal variations as a function of the examined factors (e.g., irrelevant context increased the N400-amplitude). The N400 for metaphors has been associated with the cognitive effort needed to comprehend the metaphor, e.g., the search in semantic space (cf. Coulson and van Petten, 2002). In contrast, the studies reported mixed results with respect to later ERP components. The ERP results by Coulson and van Petten (2002), De Grauwe et al. (2010) and Resta (2012) revealed a more pronounced positive deflection for metaphors. Resta linked the Late Positivity to a pragmatic processing stage, which follows semantic processing (N400). Coulson and van Petten (2002) interpreted this effect in terms of recovery of the underlying conceptual metaphor. De Grauwe et al. (2010) considered demands from conflict resolution or selection of the contextually appropriate meaning. Given that late positive effects are observed outside of metaphor processing as well—in other non-literal cases, but also in semantic reversal anomalies (e.g., Regel et al., 2011; Brouwer et al., 2012; Schumacher, 2014)—a more general account of the underlying processes is warranted, reflecting resolution of conflicts from prior processing streams. Other studies on metaphor did not report later effects (cf. Pynte et al., 1996; Coulson and van Petten, 2007 and Lai et al., 2009), which could be due to the selection of the time window of interest (Coulson and van Petten, 2007) or the fact that different word classes (adjectives and verbs) were measured (Lai et al., 2009), which could point toward distinct degrees of sensibility of ERPs to different word classes and related mechanisms.

In general, the findings indicate that figurative language processing exerts costs relative to the processing of more literally used expressions, which is measurable in two discrete processing stages reflected by N400 and Late Positivity effects. However, previous ERP data cannot shed light on the time-course and contribution of literal meaning aspects, as they do not allow to tap into very early processes or to determine whether there is a mandatory initial stage of literal analysis (Bambini and Resta, 2012). A more refined method is required to address this issue. In previous behavioral studies, metaphors were already investigated through priming experiments, for instance in contextual priming studies (Gildea and Glucksberg, 1983; Glucksberg et al., 2001) or in cross-modal priming (cf. Blasko and Connine, 1993; Rubio Fernández, 2007). These priming studies showed the influence of contextual cues and the time-course of property suppression and enhancement. The cross-modal priming data by Rubio Fernández (2007) revealed priming of contextually relevant and irrelevant (literal) meaning aspects until 400 ms after the metaphor (e.g., *plant* and *spike* primed *cactus* in *John doesn't like physical contact. Even his girlfriend finds it difficult to come close to him. John is a cactus*.); 1000 ms after the critical word, the literal meaning was no longer activated. Yet, findings were mixed (cf. also Rubio Fernández, 2007) and the material used was heterogeneous (e.g., adjectives vs. nouns as primes; a mix of hyponymical, heteronymical, and meronymical prime-target relations; metaphor and metonymy interspersed). Furthermore, an even more time-sensitive method than the measure of reaction times is required to answer the question about the role of literal meaning in the early processing of figurative language more adequately. Therefore it seemed fruitful to combine the masked priming paradigm with ERPs.

### Rationale of the present study

Here we combined the highly time-sensitive method ERP with masked priming. In contrast to the reaction-time studies mentioned above, we presented the prime word immediately before the target word at which point a figurative reading emerges and time-locked the ERP to the word-recognition point of the critical word (see below for further details). Furthermore because we were interested in early automatic processes of figurative language processing, we used pattern masked priming (cf. Kiefer and Spitzer, 2000). Holcomb and Grainger (2006, 2007) provide a detailed description of the interaction of masked priming and ERPs. In this model, processing difficulties at the semantic level are primarily reflected in the N400, where the semantic meaning of the whole word is computed and therefore unrelated prime-target pairs elicit the largest amplitude followed by semantically related prime-target pairs (cf. e.g., Holcomb et al., 2005; Kiyonaga et al., 2007—but note that effects of lexical processing as early as 200 ms after stimulus onset have been reported; e.g., Pulvermüller et al., 2001 for face-related activity verbs; Kissler et al., 2007; Ponz et al., 2014 for processing of emotional information). Based on these findings from word list presentation, we successfully tested the applicability of the masked priming ERP paradigm to sentence processing (Schumacher et al., 2012). Using a procedure as described in more detail in Procedure and illustrated in **Figure 2**, participants listened to sentences for comprehension (e.g., *A student attended a talk in Berlin*) and looked at a pattern mask display. 100 ms before the target word (e.g., *talk*), a masked word was presented visually for 67 ms. ERPs time-locked to the recognition point of the target word revealed that a related prime (e.g., *speaker-talk*) engendered a lower N400-amplitude relative to an unrelated prime (*tailor-talk*) in sentential context but also in word lists, reflecting facilitation. This allows us to look at the role of literal meaning aspects in figurative processing by presenting a probe word associated with the literal meaning prior to the vehicle (e.g., *these songs are drugs: illegal-drugs*). Accordingly, unrelated meaning aspects should hinder processing and induce a more enhanced N400, while related meaning aspects should show facilitation. Within this paradigm, we capitalize on the N400's contribution to lexical access. Crucially, the N400 has also been associated with further subcomponents of lexical processing (i.e., storage, retrieval, integration), which are subserved by distinct neuroanatomical regions (cf. Lau et al., 2008).

**Figure 1:**
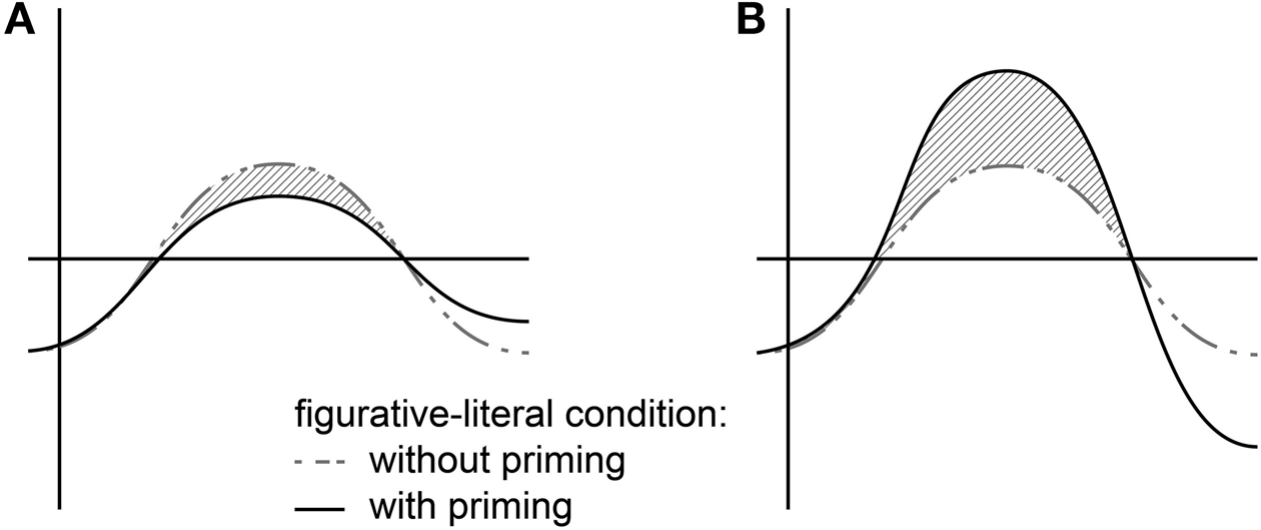
**Predictions for difference wave plots (figurative condition minus literal meaning) for condition with (gray dotted line) and without priming (black solid line) in the N400 time-window**. Left panel **(A)** illustrates the indirect access view, right panel **(B)** the direct access view.

Based on the theories discussed above and previous findings, the following predictions can be formulated for metaphor comprehension: first, we expect a biphasic N400-Late Positivity pattern with greater amplitude deflections for the metaphorical condition relative to the literal control in a comprehension task without priming (cf. Coulson and van Petten, 2002; Arzouan et al., 2007; Resta, 2012). Second, to address the question of what role the literal meaning plays in figurative language, we employ the masked priming ERP technique. This should reveal whether a probe word associated with the literal meaning eases or hinders comprehension of the vehicle, where facilitation should be reflected in reduced N400-amplitudes. To this end we also used difference wave plots to compare metaphor comprehension processes with and without priming. Difference waves are created by subtracting the literal condition from the metaphorical one for the presentation without and with priming separately. The hypotheses for the comparison of the factor priming are schematically illustrated in Figure 1. The indirect access approach (Grice, 1975; Searle, 1979) and also the theories by Recanati (1995), Giora (1997), and Carston (2002) predict the literal prime to have no negative and even a facilitating effect on the computation of both conditions, since the property of the literal meaning counts as a related prime for these accounts. Hence with priming, the N400-amplitude difference between literal and figurative conditions should remain the same or even decrease if the prime has a more positive impact on the figurative condition, as can be seen in Figure 1A. In contrast, the direct access approach and parallel or relevance-theoretic approaches (e.g., Gibbs, 1989; Kintsch, 2000; Glucksberg, 2008; Sperber and Wilson, 2008) argue for a hampering effect of the literal prime in the figurative condition since the literal meaning is not accessed initially. Hence, when primed, the N400-amplitude should increase for the figurative condition and decrease for the literal condition. As a result, the difference plot for the primed conditions should show a more pronounced negativity as is shown in Figure 1B.

As a secondary goal of this research, we wanted to compare metaphor and one particular type of metonymy. This was motivated by the observation that existing theories make different proposals about the (dis)similarity of the processes underlying the computation of metaphors and metonymies. Some accounts argue for the processes to be the same (e.g., Sperber and Wilson, 1985, 2008; Frisson and Pickering, 2001), others suggest them to be different (Lakoff and Turner, 1989; Croft, 1993, 2002). The term metonymy is used for utterances in which a word or phrase is used to refer to something connected to the used expression, e.g., the name of an artist for the work produced by him (*The boy read Böll*). This close connection between the two readings may be directly reflected in the lexical representation (cf. e.g., Pustejovsky, 1995; Asher, 2011). Furthermore, different underlying mechanisms have been ascribed to a range of metonymy types (e.g., Copestake and Briscoe, 1995; Nunberg, 1995). For example, producer-for-product metonymy is less context-specific, frequently used and based on general patterns like “X for Y”. In contrast, cases like *The ham sandwich wants to pay* are categorized as meaning transfer, resulting in a reconceptualization of the source. For the cognitive linguistic approach, metonymy is based on mapping within a domain or domain matrix (cf. Lakoff and Turner, 1989; Croft, 2002) or represents a conceptual shift (cf. e.g., Barcelona, 2002; for an overview see Panther and Thornburg, 2003), whereas metaphor is subject to mapping processes between two unrelated domains (cf. e.g., Langacker, 1987; Lakoff, 1993). Accordingly, in *The boy read Böll*, *Böll* relies on a domain that includes the concepts “person” and “work of Böll.”

The comprehension of producer-for-product metonymy has been investigated behaviorally, indicating no processing effort (Bambini et al., 2013; see Frisson, 2009 for an overview). While this type of metonymy has not been tested using ERPs before, there are a number of existing studies on logical metonymy (*The boy began the novel*) and different types of nominal metonymies (content-container alternations: *Tim put the beer on the table*; *Tom drank the bottle*), including reference transfer like *The ham sandwich wants to pay* (Kuperberg et al., 2010; Schumacher, 2011, 2013, 2014). These studies cannot support a unified account for processing metonymy. They suggest that metonymies that can be resolved by meaning selection in the lexical representation evoke an N400 and that meaning adjustment that requires reconceptualization (and hence modification of discourse representation) engenders a Late Positivity (cf. Schumacher, 2013). By testing producer-for-product metonymies we want to contribute to this typology and also establish the link to metaphor processing. Using masked priming can provide further insights into the role of literal meaning components.

## Experiment 1—literal meaning in metaphor comprehension

In this experiment, we compared the processing of nominal metaphors with that of literal expressions in German to investigate the time-course of metaphor comprehension and whether the literal meaning of a word is activated in the processing of a metaphor. First, the methods applied in the experiment without and with literal primes are described. Then, the results for metaphor without (Experiment 1a) and with priming (Experiment 1b) are reported and finally compared with respect to the impact of priming. Experiment 1a and 2b and Experiment 1b and 2a were presented together in one session but for expository reasons, we presented them as Experiment 1 and 2 separately.

### Methods

#### Participants

In total, 56 right-handed native speakers of German were paid for participating in this study. All reported normal or corrected-to-normal eyesight and no history of neurological disorder. 27 took part in Experiment 1a. Due to too many artifacts from eye-movement, three of them had to be excluded from the data analysis; hence 24 participants entered the statistical analysis (mean age 25.1, ranging from 20 to 30, 17 female). In Experiment 1b, four of the 29 participating subjects had to be excluded from the data analysis because of extensive ocular artifacts. Therefore 25 participants (mean age 24.2, ranging from 19 to 29, 15 female) entered the analysis of the ERP data.

#### Stimuli

The stimuli were carefully controlled for several factors that are known to influence the processing of metaphors and in particular the N400 effect. First, we collected the familiarity values (cf. Pynte et al., 1996; Lai et al., 2009) and chose metaphors that are neither already lexicalized nor completely unfamiliar (using a scale from not known (1) to well known (5), metaphors from the middle range were selected—see Table 1 for values). Second, since Kutas and Hillyard (1980) showed that senseless sentences elicit a more pronounced N400 than meaningful utterances, we asked participants to judge the sensicality of the metaphors and the respective literal control sentences. Third, another factor that is known to influence the N400 is cloze probability. More expected words (high cloze probability value) elicit a reduced N400 in contrast to less expected ones (cf. Kutas and Hillyard, 1984). Therefore we truncated the sentences before the critical word and asked participants to complete the sentence fragments by writing down the first continuation that came to their mind. These completions were compared with the actual sentence endings and the percentage of accordance was calculated for each item (regular cloze probability). We also employed a novel approach by analyzing the completions on the basis of whether they resulted in a metaphorical or a literal reading (category cloze probability). This second step was guided by the idea that based on theoretical approaches that focus on type-mismatches (e.g., Pustejovsky, 1995; Asher, 2011) or processes within or between domains (e.g., Croft, 2002), it seems promising to determine categorical expectations as well, in order to test whether the N400 is sensitive to category-specific (±metaphorical) predictions of the processor. For that reason, we also calculated the values of categorical accordance by counting the category matches, i.e., metaphorical completions for the metaphorical items and literal completions for the literal items. Based on these pre-test, we selected 40 metaphors and corresponding control sentences whose values are summarized in Table 1.

**Table 1:** **Summary of mean values from pre-tests for selected metaphors and corresponding literal controls**.

**Condition**	**Familiarity (*SD*)**	**Sensicality (*SD*)**	**Cloze probability**	**Category cloze probability**
Metaphor	3.05 (1.38)	3.14 (1.37)	0.0%	0.7%
Literal control	no value	1.68 (1.01)	0.0%	99.8%

Familiarity and sensicality were rated on five-point scales. For familiarity, the endpoints were labeled not known (value = 1) and well known (value = 5). In the sensicality rating, a happy smiley stood for meaningful (value = 1) and a sad smiley for meaningless (value = 5). The term category refers to figurative or literal continuations.

To summarize, the 40 chosen metaphors received medium familiarity scores, to assure that no dead (lexicalized) or totally unknown metaphors were used. The literal controls and metaphors do not differ with respect to their cloze probability values, but with respect to the categorical completions (category cloze probability). As can be seen, the metaphors were classified between meaningful and meaningless whereas the literal controls were rated more toward the meaningful endpoint of the scale. This is a typical pattern in the metaphor literature (see also the material in Bambini et al., 2013). Crucially, the selected metaphors were not rated as anomalous or meaningless. Example stimuli are provided in Table 2.

**Table 2:** **Example of critical stimuli for Experiment 1a and 1b**.

**Condition**	**Stimuli**	**Prime**
Metaphor	Diese Lobbyisten sind Hyänen, wenn man der Erzieherin glaubt.	fellig
	These lobbyists are hyenas, if you the kindergarten teacher believe.	
	*These lobbyists are hyenas, if you believe the kindergarten teacher.*	*furry*
Literal control	Diese Raubtiere sind Hyänen, wenn man der Erzieherin glaubt.	fellig
	These carnivores are hyenas, if you the kindergarten teacher believe.	
	*These carnivores are hyenas, if you believe the kindergarten teacher.*	*furry*

In the conditions with priming (Experiment 1b), the critical word (target; e.g., *hyenas*) was primed with a property of the literal meaning of the target, e.g., *furry.* Based on the close linkage between concepts and their properties (cf. Solomon and Barsalou, 2004) and to avoid problems with potential differences in the relation between prime words and targets (cf. Becker, 1980; Rubio Fernández, 2007), we only used adjectives as primes that were identified as properties of the literal and not of the figurative meaning of the corresponding target word. The appropriate properties were identified in a pre-test, in which participants saw a noun (*hyena*) and a property (*furry*) and had to rate the coherence between these two. Each noun was presented three times with different preselected adjectives to identify the one with the highest coherence value. The summary of the property values can be seen in Table 3. Since we used the same word as prime for the literal and the figurative condition, no confounding effects due to the range are expected. See the supplementary material for the whole set of stimuli (target, vehicle, and prime) and respective property coherence values.

**Table 3:** **Summary of results from pre-tests for selected primes for Experiment 1b**.

	**Word length (range)**	**Syllables (range)**	**Word Frequency (range)**	**Coherence (*SD*)**
Prime	7.13 (4–10)	2.08 (1–3)	15.39 (6–24)	5.42 (0.82)

Frequency values are based on wortschatz.uni-leipzig.de. Coherence was assessed on a six-point scale from no coherence (1) to strong coherence (6) between the target word and a particular property.

In Experiment 1a and 1b, the 80 critical sentences were presented together with 208 filler sentences in three different pseudorandomized orders. The sentences were recorded as natural speech by a female German native speaker in a sound-attenuated booth. Phonetic analyses of the critical targets (targets) and comparisons of duration, pitch and intensity registered no significant differences between the conditions (all *F*s < 1).

#### Procedure

**Figure 2:**
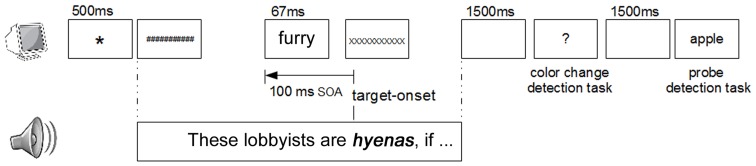
**Cross-modal masked priming procedure**. Schematic illustration of the priming procedure in sentential context.

We used a cross-modal masked priming paradigm adopted by Kiyonaga et al. (2007) and verified in Schumacher et al. (2012) in which the targets were part of auditorily presented sentences, as can be seen in Figure 2. Since priming was set as a factor, sentences for Experiment 1a and 2a were presented without primes (but with the forward mask on display) and stimuli for Experiment 1b and 2b with the masking procedure. We now explain the latter in more detail. A fixation asterisk was presented at the beginning of each trial for 500 ms in the center of the monitor. It was followed by the forward mask that consisted of 11 hash marks (#) and the auditory stimulus that started simultaneously. In the condition with priming, the forward mask was replaced by the prime 100 ms before the onset of the auditorily presented target word, hence with 100 ms stimulus onset asynchrony (SOA). The prime was presented for 67 ms and then immediately replaced by the backward mask that consisted of 11 capitalized “X.” Until the end of the auditory stimulus, the backward mask remained on the monitor. The sentence presentation was followed by a 1500 ms long blank screen and then by a question mark. At this point, participants had to perform the first of two tasks, which we employed to control for their attention. This first task (color change detection) controlled for the attention paid to the visual display and additionally was meant to distract the participants from the prime presentation. Participants had to detect a color change in the pattern masks (in 44% of all trials), which lasted for only 100 ms. The color change occurred on the forward mask, at least 1000 ms before the target, to avoid an impact on the recorded critical interval. The first task ended by participants pressing one of two buttons (“Yes” or “No”) with a maximum response latency of 2000 ms. Following another blank screen of 1500 ms, the second task (probe recognition), implemented to force the participants to pay attention to the auditory stimuli, was indicated by a visually presented word. Participants had to determine whether they had heard this word in the preceding sentence or not. The pressing of one of two possible answer buttons terminated the trial that was followed by a 1500 ms long blank screen. After that, the next trial started. The visual stimuli were presented in the middle of the screen in off-white against a black background. The letters were shown in Deja Vu Sans Mono font (34 pt.), in which all letters have the same width.

Before each session, participants were carefully instructed about the task. The main experiment was divided in eight blocks with short pauses in-between and preceded by a short training block. Afterwards, a prime detection task was administered to assess the individual prime awareness (cf. Kiyonaga et al., 2007), by first asking the participants in an informal manner if they had recognized anything outstanding. Second, after being informed about the masked priming shortly, the participants saw 30 primes under similar visual conditions (the forward mask lasted 933 or 1933 ms, the prime was again presented for 67 ms and the backward mask lasted 1000 ms) but without auditory stimuli. During the experimental session, the participants sat in front of a 17-inch monitor in a soundproof cabin.

#### EEG recording procedure

We recorded the electroencephalogram (EEG) from 26Ag/AgCI scalp electrodes mounted on the scalp by an elastic cap (*Electro-Cap International*). The EEG was digitized at a rate of 500 Hz and amplified by a *Brain Vision Brain-Amp* amplifier: impedances were kept below 4 kΩ. The EEG was referenced online to the left mastoid and re-referenced offline to linked mastoids. We placed the ground at AFz, three electrodes around the subject's right eye (over and under the eye and at its outer cantus) and one electrode at the outer cantus of the left eye. The eye-electrodes served to control for artifacts from eye-movement. To avoid slow signal drifts, the EEG data were processed offline with a 0.3–20.0 Hz band pass filter.

Crucially, previous auditory studies reported that the ERP signature, in particular the N400, varied depending on the word recognition point (cf. van Petten et al., 1999; O'Rourke and Holcomb, 2002; Schumacher et al., 2012). When time-locked to word onset, there were N400-differences for words with early and late word recognition points. When time-locked to the word recognition point, these differences diminished. Therefore we determined the word recognition point of each critical target in a gating task and time-looked the ERPs to it. For the gating task, the critical words were cut individually and then judged by six native speakers of German that were asked to listen to each sentence carefully and to identify the target word by completing it verbally. By extending the sentences in 50 ms steps, we determined the point at which most participants were able to correctly identify the target word. The word recognition point was on average 168 ms (range 24–374 ms) after the word onset.

Average ERPs were calculated per condition, participant and electrode from the word recognition point to up to 1500 ms and then subjected to automatic (rejection criterion of EOG: >40 μ V) and manual rejections. 17.37% of all trials had to be excluded due to artifacts. Because of false responses in the probe task or time-outs, 4.97% of the trials were also excluded. In total, 71.4% of the trials without priming (Experiment 1a) and 80.8% of the trials with priming (Experiment 1b) entered the statistical analysis.

#### Data analysis

We ran statistical analyses for the behavioral data over accuracy rates and reaction times over subjects and items for both tasks. The critical time-windows were predefined by visual inspection. ANOVAs of the ERP data were computed with the factor FIGURATIVENESS (figurative vs. literal) and the factor ROI (topographical region of interest), computed for lateral and midline channels separately. The lateral electrodes were grouped by location as follows: left anterior (F7/F3/FC5/FC1/C3), right anterior (F4/F8/FC2/FC6/C4), left posterior (T7/CP5/CP1/P7/P3), and right posterior (T8/CP2/CP6/P4/P8). The six electrodes form the midline were grouped pair-wise: frontal (Fz/FCz), central (Cz/CPz), and parietal (Pz/POz). Only trials with correct responses to the probe recognition task entered the analysis. The statistical analyses were carried out in a hierarchical manner. To control for potential type I errors due the violations of sphericity, the data were adjusted using the Huynh-Feldt procedure (cf. Huynh and Feldt, 1970).

### Experiment 1a

#### Behavioral results

For the color change detection and the probe recognition task, we calculated reaction times and accuracy rates for the literal and metaphorical condition separately. With over 94% correct answers, the results revealed that the participants paid attention to the visual (94.81%, *SD* = 0.12) and auditory (94.86%, *SD* = 0.03) stimuli. Statistical analyses revealed no differences for the factor FIGURATIVENESS for accuracy rates and reaction times in both tasks (all *F*s < 1).

#### Electrophysiological results

**Figure 3:**
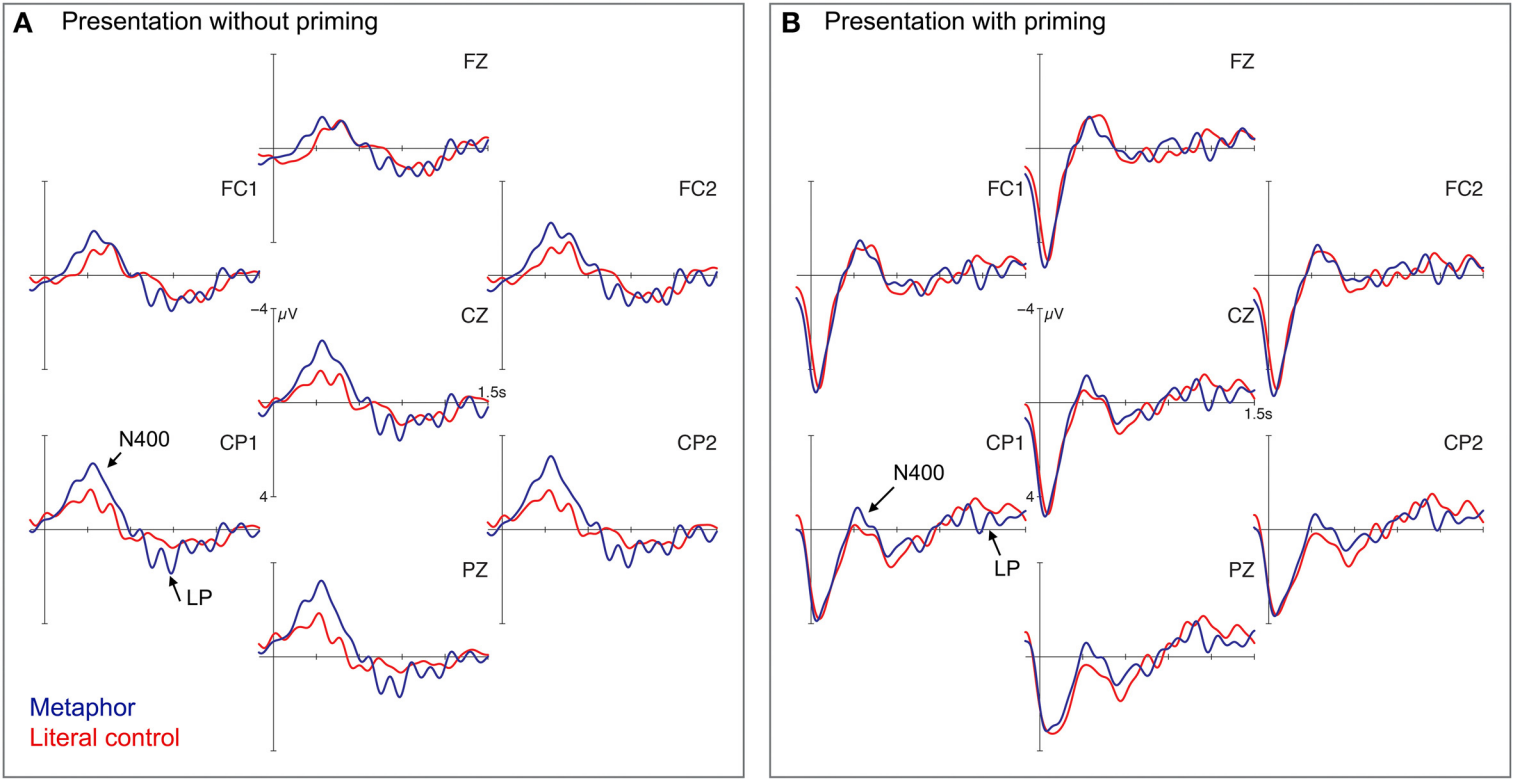
**Grand average ERPs for Experiment 1a and 1b**. Grand average ERPs for 7 selected electrode sites for metaphorical (blue) and literal (red) conditions without priming in **(A)** (Experiment 1a) and with priming in **(B)** (Experiment 1b). Negativity is plotted up. Vertical bar represents the word recognition point of the target.

Visual inspection of the grand average ERPs revealed two critical time-windows for the comparison of the literal and the figurative condition (see Figure 3A): a more negative deflection for metaphors between 250 and 500 ms (N400-window) and a more positive deflection between 700 and 900 ms (Late Positivity). We ran separate ANOVAs for both time windows that revealed an interaction of FIGURATIVENESS × ROI between 250 and 500 ms (N400 time-window) [*F*_(3, 69)_ = 13.67, *p* < 0.001], significant in the left [*F*_(1, 23)_ = 6.86, *p* < 0.05] and right [*F*_(1, 23)_ = 18.12, *p* < 0.001] posterior regions. For the midline electrodes, the statistical analyses for the N400-window also revealed an interaction of FIGURATIVENESS × ROI [*F*_(2, 46)_ = 17.25, *p* < 0.001], significant in the central [*F*_(1, 23)_ = 10.29, *p* < 0.01] and posterior [*F*_(1, 23)_ = 17.64, *p* < 0.001] regions. For the Late Positivity time-window (700–900 ms), ANOVAs showed an interaction of FIGURATIVENESS × ROI for the lateral electrodes [*F*_(3, 69)_ = 3.48, *p* < 0.05] significant in both left [*F*_(1, 23)_ = 18.37, *p* < 0.001] and right [*F*_(1, 23)_ = 13.33, *p* < 0.01] posterior regions. For the midline electrodes, statistical analyses also revealed a main effect of FIGURATIVENESS in the late time-window [*F*_(1, 23)_ = 12.31, *p* < 0.01].

#### Discussion

Experiment 1a investigated the processing of nominal metaphors in German without priming. ERPs revealed a biphasic N400-Late Positivity with more pronounced deflections for the metaphorical in comparison to the literal condition. The behavioral data indicated high attentiveness of the participants to visual and auditory stimuli.

The ERP data can be interpreted in line with the idea that the N400 reflects enhanced costs in the lexical access phase, influenced by context and the degree of categorical expectancy. The classical cloze probability values are at 0% in both conditions, therefore the N400-difference (250–500 ms) cannot be explained based on the expectancy of a particular word. The category expectancy value however matches the N400 deflection. The high expectation of any word that completes the sentence literally (almost 100%) elicited a less pronounced N400-amplitude than the unexpected metaphorical completion with any word belonging to the metaphorical category (value of categorical accordance below 1%). In the later time-window, metaphors showed a more pronounced positive-going wave between 700 and 900 ms than the literal control condition. This Late Positivity might be interpreted as reflecting enhanced costs due to pragmatically or inferentially driven mapping processes, involving two unrelated domains. This has consequences for discourse representation: in the metaphorical condition, the integration of a referent in the discourse involves the combination of two domains and processing is hence more costly than the simple establishment of a new referent in the literal condition.

Experiment 1a, like other studies before, found enhanced costs for the processing of metaphors in comparison to literal utterances. Hence, theories that argued for metaphors to be interpreted as easily as comparable literal utterances are challenged (e.g., Sperber and Wilson, 1986; Glucksberg, 2008). In contrast, these findings support theoretical accounts that assume more steps or higher effort during the interpretation of metaphors (cf. Grice, 1975; Searle, 1979; Carston, 2010b). Yet these results do not allow to discriminate between different accounts on the steps in figurative processing.

For the reason that we are interested in the role of literal meaning during the early processing stage in the computation of metaphors, Experiment 1a served to set up a baseline. In the following, the same materials were presented with primes that were literal properties of the critical word and then the results are compared with the findings for metaphor processing without priming.

### Experiment 1b

#### Behavioral results

As before, participants performed well in both tasks, indicating that they paid high attention to the visual and auditory stimuli. They responded correctly to 98.6% (*SD* = 0.05) of the color change detection and 95.9% (*SD* = 0.02) of the probe recognition task. For accuracy rates, ANOVAs with the factor FIGURATIVENESS revealed no significant differences for both tasks (all *F*s < 1). For reaction times, statistical analyses showed no differences for the color change detection task (all *F*s **<** 1) and for the probe recognition a significant differences for the subject analysis only [*F*_1(1, 24)_ = 5.13, *p* < 0.05; *F*_2_ < 1]. This was due to slower reaction times for the literal (mean = 909 ms) than for the figurative condition (877 ms).

#### Electrophysiological results

Figure 3B shows the grand average ERPs in the masked priming paradigm. To allow for a good comparison with the unprimed conditions, we picked the same time-windows between 250 and 500 ms (N400) and 700 and 900 ms (Late Positivity). Crucially, the figurative and the literal conditions did not seem to differ in this Late Positivity-window but further downstream. Indeed, statistical analyses showed no significant effect for the 700–900 ms time-window (*F* < 1). For the N400 time-window, ANOVAs revealed an interaction of the factors FIGURATIVENESS × ROI [*F*_(3, 72)_ = 7.71, *p* < 0.001], which was resolved significantly in the left [*F*_(1, 24)_ = 6.92, *p* < 0.05] and right [*F*_(1, 24)_ = 7.05, *p* < 0.05] posterior regions, and for the midline electrodes [*F*_(2, 48)_ = 10.03, *p* < 0.001], significant in the posterior region [*F*_(1, 24)_ = 4.82, *p* < 0.05]. Additionally, we analyzed the time-window between 1100 and 1300 ms (based on visual inspection) in which the metaphorical condition elicited a more positive deflection than the literal control condition. ANOVA showed a main effect of FIGURATIVENESS for the lateral [*F*_(1, 24)_ = 5.49, *p* < 0.05] and the midline electrodes [*F*_(1, 24)_ = 4.41, *p* < 0.05].

#### Post-ERP test

As described in *Subsection* Procedure, participants were asked to perform a prime detection task. On average, participants detected 18 of 30 primes correctly. The average prime detection rate hence was 59.3%, which mirrors the results from other experiments (e.g., Kiefer, 2002; Kiyonaga et al., 2007). In addition, we controlled for a possible influence of the individual prime detection rate on the size of the N400. Therefore we calculated the correlation of the prime detection rate and N400-amplitude difference for three midline electrodes separately (Cz, CPz, and Pz) by subtracting the maximal amplitude value in the critical time-window (250–500 ms) of the literal from that of the figurative condition for each participant. These values were then correlated with the individual prime detection rates. The statistical analysis revealed no reliable correlation for any of the three electrodes: Cz (Pearson's *r* = 0.111, *p* = 0.598), the CPz (*r* = 0.161, *p* = 0.442) or the Pz (*r* = 0.167, *p* = 0.425).

#### Discussion

In this experiment, metaphors were presented within a masked priming paradigm to investigate the role of literal meaning during the lexical access phase of the critical word. As in the condition without priming, we found a more pronounced N400^3^ (250–500 ms) and Late Positivity (1100–1300 ms) for the metaphorical in comparison with the literal condition. Hence, independent of priming, the processing of the critical word (*hyenas*) is more demanding in the metaphorical environment than in the literal during the lexical access phase as well as in later discourse updating processes.

**Figure 4:**
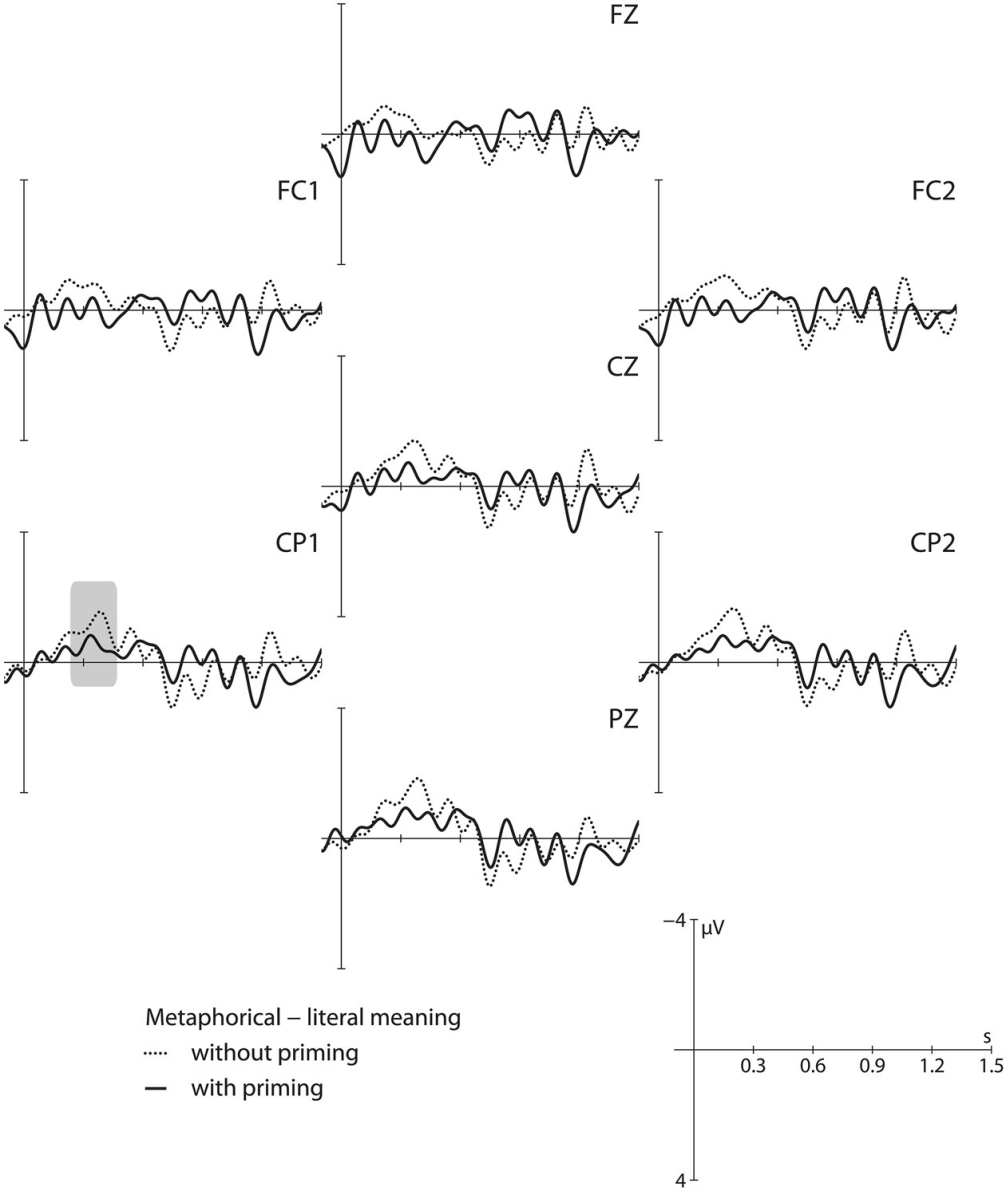
**Difference wave plots for Experiment 1**. Difference waves for metaphorical minus literal condition without (dashed line) and with (solid line) priming. The vertical bar marks the word recognition point of the target. The critical time-window is shaded in gray.

To see in which direction the literal primes influence the N400 (de- or increasing amplitude-difference), we calculated difference wave plots by subtracting the literal from the metaphorical condition for both experiments (without and with priming) separately (cf. Roehm et al., 2007). This allowed us to filter out differences between the two participant groups and differences arising from the different presentation modalities. Visual inspection of Figure 4 revealed a slightly reduced N400-amplitude difference (between 250 and 500 ms) for the presentation with a literal prime in Experiment 1b. This was supported by statistical analyses (*p*'s < 0.01). The literal prime word therefore has a facilitating effect on language processing (cf. Rolke et al., 2001; Kiefer, 2002; Grossi, 2006). The fact that the N400-amplitude difference in the masked priming conditions is even reduced indicates a greater benefit of the literal prime word in the figurative than in the literal interpretation. Processing the metaphor may profit from the subliminal prime due to pre-activation of the semantic network of the target, which eases the extra operations required. The data suggest that the pre-activation of the literal meaning of the target word within a metaphor does not hamper, but rather facilitates processing.

Based on these observations, accounts that maintain access to literal meaning aspects like the indirect access view (Grice, 1975; Searle, 1979) are supported. Likewise, theories that expect the literal meaning to linger around in figurative language processing are substantiated as well (Giora, 2008; Carston, 2010b). In contrast, theories that claim that the literal meaning does not play a role in metaphor processing (Sperber and Wilson, 2008) or that predict a hampering effect of the literal prime word (Glucksberg, 2008) cannot be confirmed by the current results. The difference wave plots thus reveal evidence against the direct access account.

Beside the reduced N400-amplitude, priming had an impact on later components. With priming the Late Positivity was delayed from 700–900 ms to 1100–1300 ms. The latency shift might result from interferences from the prime presentation in earlier phases that hamper (later) pragmatic operations. A possible explanation might be that this delay is caused by the literal prime holding up the mapping or reconceptualization processes. Note however that the previous ERP experiment on priming in sentential context already revealed an influence of masked priming in later processing stages where the repetition priming condition registered a late positive deflection in sentential context but not in list presentation (cf. Schumacher et al., 2012). To decide whether the linguistic information of the primes or a more general disturbance triggered by prime presentation provoke the latency shift, further investigations are needed.

In sum, metaphors elicited a more pronounced biphasic N400-Late Positivity pattern for the metaphorical condition in comparison with the literal controls independent of prime presentation. The processing of metaphors therefore can be interpreted as more costly than the processing of literal language. When preceded by a literal prime word, the effort in the lexical access phase of the target word is reduced (smaller amplitude difference) and the Late Positivity is delayed. The reduced N400 refutes the direct access view and supports the idea that the literal meaning is accessed or at least lingering during figurative language processing.

## Experiment 2—literal meaning in metonymy comprehension

To extend the findings for the role of literal meaning to another type of non-literal language, we also tested producer-for-product metonymies utilizing the same experimental design as for metaphors.

### Methods

#### Participants

We gathered data from the same 56 participants as tested in Experiment 1. In Experiment 2a, five of 29 participants had to be excluded due to too many artifacts; hence 24 subjects (mean age 24.3, 15 female) entered the analysis of the ERP data. In Experiment 2b (with priming), the ERP data of 22 subjects (mean age 24.9, age ranged from 21 to 30, 16 women) were analyzed after discarding data from five participants due to extensive ocular and motion artifacts.

#### Stimuli

The materials were pretested on sensicality, familiarity, cloze probability, and category cloze probability. Since the tested metonymies all belong to the conventional producer-for-product type, we controlled for familiarity of the famous person used (cf. Frisson and Pickering, 2007). Therefore we conducted a test similar to the familiarity pretest by Frisson and Pickering (2007) and calculated the percentage of participants that correctly named the profession of the respective famous individuals. The findings are summarized in Table 4.

**Table 4:** **Summary of mean values from pre-tests for selected metonymy and corresponding literal controls**.

**Condition**	**Familiarity**	**Sensicality (*SD*)**	**Cloze probability**	**Category cloze probability**
Metonymy	90.52%	1.58 (0.88)	0.2%	2.8%
Literal control		2.02 (1.21)	0.0%	99.6%

Familiarity reports the percentage of participants who identified the correct profession for the famous people used in this type of metonymy. Sensicality was assessed on a five-point scale ranging from meaningful (value = 1) to meaningless (value = 5). Category refers to figurative or literal continuations.

Again it was necessary to find good primes for the literal meaning of the target word (all last names of famous people like *Böll*). We used the same property acceptability test as described for metaphors and used adjectives as properties to keep the conditions similar to the metaphor experiment. Since we controlled for the fact that the properties should not represent potential properties of the metonymical meaning, it was challenging to find appropriate properties. Many adjectives, e.g., *lively*, that describe famous individuals (as e.g., painters) also describe their work, i.e., the metonymical meaning. Therefore, we used rather general adjectives comprising of human and biographical characteristics (e.g., *divorced, talented*). Their characteristics are summarized in Table 6. The critical words and properties with the corresponding property coherence values are added in the supplementary material.

In both Experiments (2a and 2b), the 80 critical sentences were presented together with 208 filler sentences in three different pseudorandomized orders. An example of the metonymies of the type producer-for-product (author-for-work, designer-for-clothing, composer-for-composition, and painter-for-painting) and their literal control sentences can be seen in Table 5.

**Table 5:** **Example of critical stimuli for Experiment 2a and 2b**.

**Condition**	**Stimuli**	**Prime**
Metonymy	Der Student las damals Böll bei einer Versammlung.	talentiert
	The student read at that time Böll during an assembly.	
	*At that time the student read Böll during an assembly.*	*talented*
Literal control	Der Student begegnete damals Böll bei einer Demonstration.	talentiert
	The student met at that time Böll during a protest.	
	*At that time, the student met Böll during a protest.*	*talented*

**Table 6:** **Summary of results from pre-tests for selected primes for Experiment 2b**.

	**Word length (range)**	**Syllables (range)**	**Word Frequency (range)**	**Coherence (*SD*)**
Prime	8.1 (4–10)	2.42 (1–3)	15.2 (11–23)	3.1 (1.52)

Frequency values are based on wortschatz.uni-leipzig.de. Coherence was assessed on a six-point scale from no coherence (1) to strong coherence (6) between the target word and a particular property.

The sentences of the metonymical condition were recorded by the same female German native speaker and under the same conditions outlined in Experiment 1. Again phonetic analyses of the critical targets registered no significant differences (all *F*s < 1) between the metonymical and the literal condition in terms of duration, pitch and intensity.

#### Procedure

We used the same procedure as in Experiment 1.

#### EEG recording procedure

The recording procedure was the same as in Experiment 1. Six participants determined the word recognition point for the critical targets in a gating task (on average 191 ms (ranging from 18 to 398 ms) after name onset). Because of probe task responses and filtering procedures, we had to exclude 7.78% of the trials due to artifacts and incorrect responses for the condition without priming and 8.49% of the trials for the condition with priming.

#### Data analysis

ANOVAs were carried out for the behavioral data over reaction times and accuracy rates for both tasks. The critical time-windows for the ERP analysis were determined by visual inspection and statistical analyses were computed for the mean amplitude value of the ERP data. ANOVAs for Experiment 2a (without priming) and 2b (with priming) were calculated with the factor FIGURATIVENESS (figurative vs. literal) and ROI (topographical regions of interests).

### Experiment 2a

#### Behavioral results

The behavioral responses indicated that participants paid attention to the visual and auditory stimuli. The color change detection task yielded over 98% (*SD* = 0.07) correct responses, the probe recognition task over 96% (*SD* = 0.02). For both tasks, ANOVAs for accuracy rates revealed no differences for the factor FIGURATIVENESS for subjects and items (all *F*s < 1). For reaction times, statistical analyses for the color change detection task also revealed no effects (all *F*s < 1) and for the probe recognition task a reliable difference by subjects only [*F*_1(1, 23)_ = 7.48, *p* < 0.05]. This was reflected in faster reaction times for metonymies (mean = 856 ms) than for the literal control sentences (mean = 885 ms).

#### Electrophysiological results

**Figure 5:**
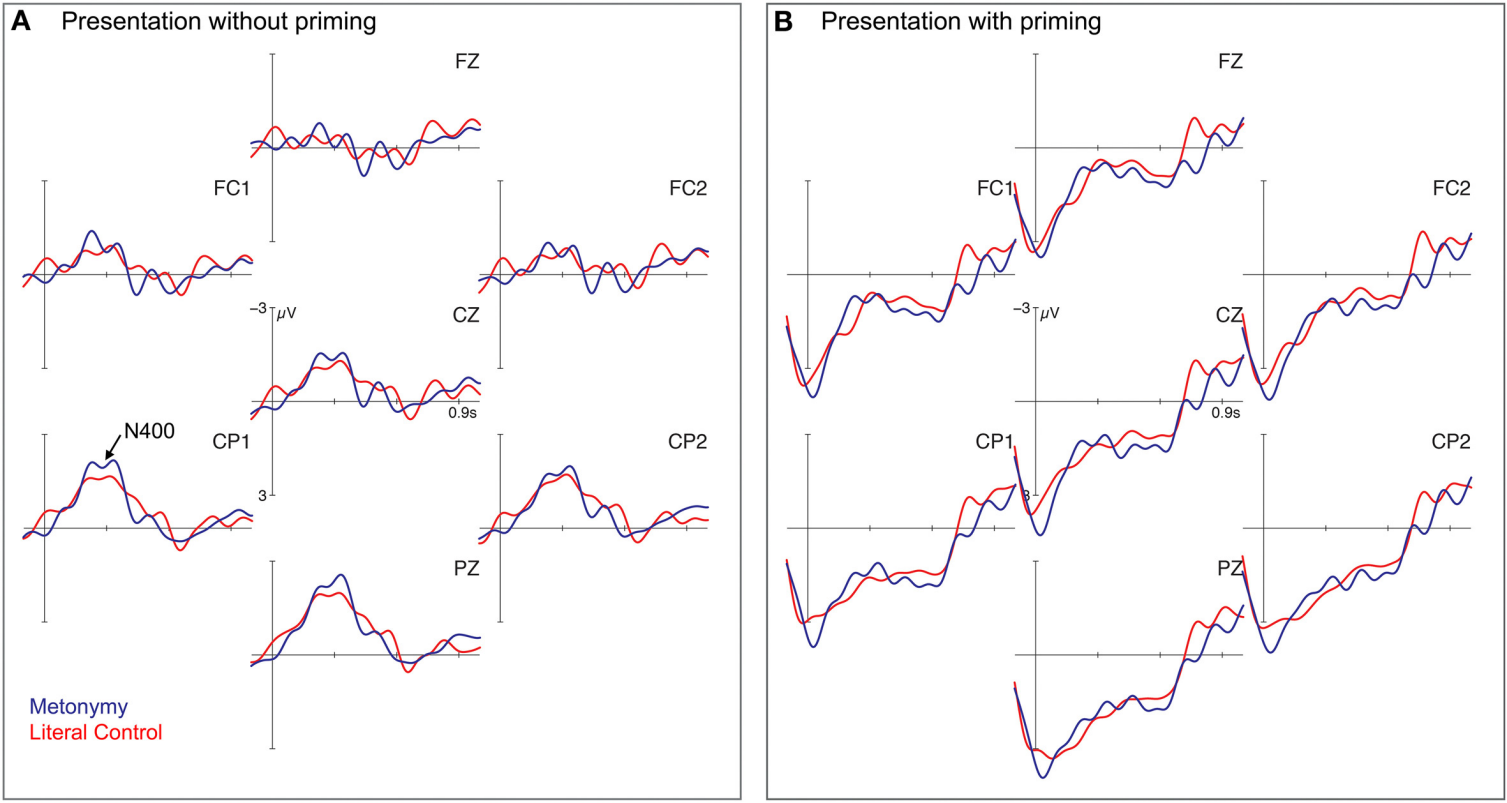
**Grand average ERPs for Experiment 2a and 2b**. Grand average ERPs at 7 selected electrodes for metonymic (blue) and literal (red) conditions without priming in **(A)** (Experiment 1a) and with priming in **(B)** (Experiment 1b). Vertical bar represents the word recognition point of the target; negativity is plotted up.

Figure 5A shows the grand average ERPs for metonymies and their literal controls. The figurative condition elicited a more pronounced negativity between 200 and 350 ms (N400 time-window) in contrast to the literal condition. The findings, based on visual inspection, were confirmed by statistical analyses. ANOVAs revealed an interaction of FIGURATIVENESS × ROI [*F*_(3, 69)_ = 3.11, *p* < 0.05], significant only in the left posterior region [*F*_(1, 23)_ = 4.45, *p* < 0.05], for the lateral regions of interest and no significant effect for the midline (*F* < 1).

#### Discussion

In this experiment, German producer-for-product metonymies were presented without priming. ERPs revealed a more pronounced N400 (200–350 ms) and no later effects^4^. This result is in contrast to previous studies on metonymy that reported a biphasic pattern (reference transfer; Schumacher, 2014) or a monophasic Late Positivity (container-for-content metonymy; Schumacher, 2013). In turn, for the more conventionalized producer-for-product metonymies tested here, eye-tracking studies (Frisson and Pickering, 1999, 2007; McElree et al., 2006) and timed sensicality judgments (Bambini et al., 2013) did not find differences between metonymical and literal utterances if the author was familiar (*read/met Dickens*). However, for unfamiliar authors the processing of the metonymical condition, assessed via eye-tracking, was more costly (*read/met Needham*) if presented without supporting context. Since we presented the metonymies with well-known famous people (familiarity value >90%), one would expect to find no differences based on the former results.

In comparison with previous studies on metonymy, we can draw two important conclusions: first, the ERP data substantiate the existence of different types of metonymy and their respective underlying processes. We assume that producer-for-product metonymy is based on a general metonymic pattern (“X for Y”). The *ham sandwich* example tested by Schumacher (2011, 2014), is categorized as a case of reference transfer since it requires transfer operations on the discourse referent. The interpretation of examples like *The ham sandwich wanted to pay* requires information that is stored in the lexical entry of a restaurant script. Since it is not expressed in the utterance, the processor has to relate these two domains (Nunberg, 1995). This differentiation can explain the findings for reference transfer and producer-for-product metonymy^5^. The producer-for-product type only requires the selection of the correct meaning from the lexical representation. The reference transfer type involves the establishment of a relation between two domains (e.g., “restaurant” and “ham sandwich”) via inferencing or mapping processes, which are reflected in the Late Positivity. This explains why we found this effect for metaphors too, but not for the producer-for-product metonymies, where no such operations are required (no Late Positivity). This explanation is also in line with the assumptions made by Schumacher (2013). She argued for reference transfer to involve a reference shift and therefore modifications in discourse structure and for producer-for-product metonymy to involve meaning selection processes only. Second, the observed N400 reflects a context-dependent, expectancy driven process (cf. e.g., Kutas and Hillyard, 1984). This view matches the results because the metonymical completion was expected neither in the metonymies tested here nor in the reference transfer examples. Additionally, Schumacher (2014) reported the absence of an N400-difference when the critical sentences are preceded by a supporting context (higher expectancies of a metonymical completion via pre-activation of e.g., the restaurant setting).

Finally, ERP and eye-tracking seems to have a different degree of sensibility to the underlying language computation processes. This might result from the distinct presentation modalities: in eye-tracking experiments, the participants are presented with the entire sentence at once, allowing them to regress to earlier parts of the utterance. In ERP studies, the participants do not have the possibility to check earlier parts since the material is presented as rapid serial visual sequences or auditorily.

The current experiment revealed evidence for different types of meaning adjustment. The simpler metonymy type (producer-for-product metonymy) elicited a monophasic N400 and differs from the more complex type (reference transfer) and metaphor, which both showed an additional Late Positivity. In Experiment 2b, we presented the producer-for-product metonymies with literal prime words that preceded the target. The comparison of Experiment 2a with 2b will then provide additional insights about the role of literal meaning during figurative language processing.

### Experiment 2b

#### Behavioral results

As in the preceding experiments, the participants performed well in all tasks. The accuracy rate elicited for the color change detection task was 95.03% (*SD* = 0.12) and for the probe detection task 95.6% (*SD* = 0.03). ANOVAs showed no significant differences for the factor FIGURATIVENESS for accuracy rates for both tasks (all *F*s < 1). For reaction times, statistical analyses registered no reliable difference for the color change detection task (*F*s < 1) and an effect in the subject analysis only for the probe recognition task [*F*_1(1, 21)_ = 5.74, *p* < 0.05; *F*_2_ < 1]. Participants reacted significantly faster in the figurative condition (mean reaction time = 905 ms) than in the literal control condition (mean = 932 ms).

#### Electrophysiological results

Although visual inspection of Figure 5B suggests no differences between the metonymical and the literal condition if preceded by a prime word, we ran ANOVAs over the same time-window (200–350 ms) as in the unprimed condition to keep the results comparable. The statistical results supported the impression gained by visual inspection. The N400 time-window revealed no significant effects (all *F*s < 1). Additionally, we computed ANOVAs with 50 ms time-windows from 0 to 1000 ms. The time-window from 750 to 800 ms showed a main effect of FIGURATIVENESS in the lateral and the midline analysis only (*F*s > 13.7, *p* < 0.01). Again, we do not consider this difference reliable, because the time-window is short and no adjacent time-windows elicited significant differences (cf. Gunter et al., 2000).

#### Post-ERP test

In the subsequent prime detection task, participants performed around chance level. On average they named 18 primes correctly (59.81%). Since ERPs did not differ in the potential N400-window (see below), we did not compute correlations for prime detection and N400-amplitude differences.

#### Discussion

In this experiment we presented the stimuli tested in Experiment 2a with a literal prime word to investigate the role of literal meaning in the processing of other types of non-literal language. With priming, producer-for-product metonymies did not elicit an N400 or any other later effects in comparison to the literal controls.

When presented without a literal prime, metonymies evoked an N400, reflecting enhanced costs in the lexical access phase of the critical word (e.g., *Böll*). The observation that metonymies did not elicit an N400-difference when preceded by a literal prime points either toward different underlying costs in early language processes in the comparison of metaphor and metonymy or toward a different degree of sensitivity to priming effects. Unfortunately, metaphor and metonymy were never directly compared in an ERP study before. The only studies carrying out direct comparison are behavioral. A reading and reaction time study by Gibbs (1990) tested how easily figurative reinstatements can be used as anaphors for literal referents that were introduced in a short story and reported faster reaction times for metaphors than for metonymies. This indicates higher processing effort for metonymy than for metaphor and contradicts our ERP findings. The pattern observed by Gibbs may however be confounded by an animacy shift for the metonymical (*poor surgeon*/*scalpel*) but not for the metaphorical reinstatement (*poor surgeon*/*butcher*). Additionally, the use of *the scalpel* seems less conventionalized then *the butcher* for referring to a poor surgeon. Since the material was not controlled for familiarity, conventionality or animacy, the longer reading times for metonymies could result from these factors. The current differences may therefore be best explained on the basis of typological differences between metonymy and metaphor. Conversely, the direct comparison in sensicality judgments in Bambini et al. (2013) revealed that the processing costs for interpreting metaphors are higher than for metonymy; in the latter case, response times equated the literal condition, in line with our ERP findings.

## General discussion

This study investigated the processing of figurative language by using ERPs in combination with masked cross-modal priming to examine the role of literal meaning aspects during the processing of metaphors (Experiment 1) and metonymies (Experiment 2). In the conditions without priming, metaphors revealed a biphasic N400—Late Positivity pattern, while metonymies evoked a monophasic N400. This suggests different underlying mechanisms for the processing of figurative language. In combination with masked priming, the data revealed facilitating priming effects of literal prime words when priming a figurative utterance and a different degree of the impact of the prime on metaphors (reduced N400; Experiment 1b) and metonymies (vanished N400; Experiment 2b).

### Metaphor processing

We tested nominal metaphors for the first time in German and replicated previous findings from studies that investigated other languages and different degrees of conventionality (cf. Coulson and van Petten, 2002; Arzouan et al., 2007; Resta, 2012). Metaphors evoked a biphasic pattern, which we interpret in terms of enhanced costs during lexical access of the critical word, e.g., *hyenas* (N400) and computational demands required for the modification of the current discourse representation (Late Positivity). The difficulties during lexical access can be explained by several factors, especially context integration. The N400-amplitude reflects the category cloze probability of almost 100% for the literal and below 1% for the metaphorical condition (where category refers to literal or figurative completions in the cloze task). The processor expected a literal completion in both conditions and therefore the metaphorical completion is unexpected and hampers the lexical access of the critical word. In ERP research on metaphors, the N400 has been shown to be sensitive to several factors that may interact with each other, e.g., familiarity (Lai et al., 2009) or the preceding context (Pynte et al., 1996). Taken together, all of these results converge for the N400 to reflect processing effort during lexical access. The N400 is thus sensitive to the preceding context, category expectancy, and the degree of conventionality.

As far as the Late Positivity is concerned, across the literature one can find many possible explanations: it could be associated with pragmatically driven implicatures as suggested by Grice (1975) (see Resta, 2012), mapping operations between two unrelated domains as proposed by the cognitive linguistic approach (cf. e.g., Coulson and Matlock, 2001; Croft, 2002; Wolff and Gentner, 2011; but see Lai and Curran, 2013, for the assignment of mapping processes to the N400), meaning construction via blending of cognitive models (cf. Fauconnier and Turner, 2002; Coulson and Oakley, 2005), the activation of secondary cognitive models (cf. Evans, 2010), associative processes as implied by Searle (1979) and Recanati (1995), or the generation of ad hoc concepts via narrowing and broadening (Carston, 2010a). It is noteworthy that the Late Positivity has not been reported in all of the previous experiments investigating metaphor processing. The mixed findings have already been attributed to differences in the design of the studies that did not report a Late Positivity, e.g., the involvement of different word classes or the analysis of smaller time-windows (see above). Yet the differences across experiments may also be due to qualitative differences in the metaphorical materials. The crucial distinction we want to point out is between verbal metaphors (cf. Lai et al., 2009; Lai and Curran, 2013) that elicited a monophasic N400 and nominal metaphors as in the current study that evoked a biphasic N400-Late Positivity pattern. This distinction would fit with the proposal that the Late Positivity reflects operations on discourse representation structure where costs accrue whenever a discourse referent is added to the discourse or must be modified (cf. Burkhardt, 2007; Schumacher, 2013). Hence, in the case of *These lobbyists are hyenas*, a discourse representation for *hyenas* is established but the metaphoric interpretation requires the extraction of certain properties. The shift from an entity denoting discourse referent to a property results in modifications in the discourse representation and referent deletion^6^.

Together with previous findings on metaphors, this study revealed that the processing of metaphorical utterances is more demanding than the processing of literal sentences. Thus, the results support theoretical accounts that argue for different processing cost (metaphor > literal utterance). This includes for example the indirect access account (cf. Grice, 1975; Searle, 1979), which postulates an additional step (more costs) in the processing of metaphors, and the idea of Relevance Theory by Carston (2010b), who argues for enhanced costs for metaphors due to the construction of a relevant ad hoc concept via narrowing and broadening. Although our discussion has started from the extreme poles of the direct and indirect access accounts as they emerge in the pragmatic literature, our findings are compatible with other proposals as well, for example the computational model by Kintsch (2000), who argued for metaphor processing to take place in three steps: first the semantic neighborhood of the vehicle is activated, then a network is created via spreading activation (cf. also Quillian, 1962, 1967), involving the target, the vehicle and the environment of the vehicle, and the size of which depends on the degree of the relation between those two. In the last step, the meaning of the metaphor is created by computing the connection between vehicle and target with the highest activation. Although Kintsch argued for no differences between the underlying processes in metaphors and literal utterances, our findings can still be accounted for with his model. First, the vehicle word is activated, which can be related to differences in the N400 time-window due to accessibility (contextual effects). In terms of spreading, a related prime word that immediately precedes the vehicle should ease activation (reduced N400). The Late Positivity can then be attributed to costs emerging from the computation of the connection between target and vehicle. If this is right, the Late Positivity might be sensitive to the semantic distance between vehicle and target word.

Accounts that argue against additional effort in the processing of metaphors cannot be supported (cf. Frisson and Pickering, 2001; Sperber and Wilson, 2008). The direct access view rejects the requirement of an additional step (cf. Gibbs, 1994; Glucksberg, 2008) based on previous findings that showed that metaphorical and literal sentences are read equally fast (cf. Blasko and Connine, 1993) and with no speed differences (cf. McElree and Nordlie, 1999). Although ERPs do not reveal the amount of involved steps in general, they clearly indicate enhanced cognitive effort for metaphors. This assumption is also supported by lower accuracy rates found for metaphors in comparison to literal strings using the SAT paradigm (McElree and Nordlie, 1999) and sensicality judgments (Bambini et al., 2013).

### Metonymy processing

The investigation of producer-for-product metonymy in ERP revealed the existence of at least two different types of metonymic processes. Producer-for-product metonymy only requires simple selection processes, which is reflected in a monophasic N400; it is more demanding than the processing of literal utterances since the metonymic completion is not the expected type of category, while the representation of the discourse referent is unaffected (no Late Positivity). In contrast, reference transfer (cf. Schumacher, 2014) revealed a biphasic pattern that reflected expectancy-based difficulties during lexical access of e.g., *ham sandwich* and thereafter modifications in the discourse representation structure via inferentially or pragmatically driven processes (see above for a more elaborate discussion). These findings support approaches that differentiate between various metonymy types, for instance metonymy requiring transfer operations on the referent and metonymy that is subjected to more general lexical operations of meaning adjustment (cf. Copestake and Briscoe, 1995; Nunberg, 1995). The present results are challenging for theoretical approaches that assume the same cognitive operation for both types of metonymy.

### Metaphor and metonymy in comparison

Additionally, the indirect comparison of metaphors (N400-Late Positivity) and producer-for-product metonymies (N400) without priming (Experiment 1a and 2a) demonstrated differences as well. This leads to some initial conclusions about the (dis)similarity of metaphor and metonymy processing. Both metaphor and metonymy registered a more pronounced N400-amplitude in comparison to their literal control conditions. We interpret the N400 to reflect enhanced costs during lexical access to the respective critical word due to the low category expectancy value for both figurative conditions (below 3%). Crucially, if presented within the priming condition, metonymy no longer elicited an N400 but metaphor still did. This could result from either different degrees of sensibility to priming or, more likely, from a different amount of costs required in the lexical access phase. Since we suggest that the N400 is not limited to reflecting distinct degrees of category expectancy, the difference between figurative types may be due to different meaning adjustment operations. Lexical access in metonymy might only require the selection of the appropriate meaning, while in metaphor lexical access of the vehicle includes the generation of a new meaning. Similar to the differentiation between reference transfer and producer-for-product metonymy, metaphors, in contrast to the metonymies tested here, requires operations on the discourse referent and hence showed a more pronounced Late Positivity.

Therefore the findings support accounts that argue for differences between metaphors (mapping between unrelated domains) and metonymies (conceptual shift within a domain (matrix)) (cf. e.g., Lakoff and Turner, 1989; Croft, 1993, 2002). Accounts that suggest the same cognitive costs for the interpretation of metaphors and metonymy are in contrast challenged by the results (cf. Sperber and Wilson, 1985, 2008; Frisson and Pickering, 2001).

Metaphor and metonymy are only two of many types of figurative language use. An important task for future research will be the development of a typology of figurative language that goes beyond these two types. Initial evidence for this comes from Schumacher (2013) who investigated different types of metonymy (but see also Ferretti et al., 2007 on proverbs, Regel et al., 2011 on irony, Vespignani et al., 2010 on idioms, among others). Note also that this study concentrated on temporal aspects of metaphor and metonymy. Obviously, research on the neuroanatomy of figurative processing is essential to complement our understanding of the language architecture but such an endeavor lies beyond the scope of the current research (see e.g., Bohrn et al., 2012; Rapp et al., 2012 for corresponding meta-analyses on figurative language).

### Role of literal meaning aspects

We presented metaphors and metonymies without and with priming to investigate the role of literal word meaning in figurative language processing. Based on previous studies (cf. Holcomb and Grainger, 2006; Kiyonaga et al., 2007; Schumacher et al., 2012), we expected that a semantically related prime that precedes the target word eases the processing of this target during the lexical access phase. This is reflected in a reduced N400-amplitude. In contrast, unrelated prime words should hamper lexical access and therefore result in a more pronounced negative-going wave. We used this knowledge to investigate two theoretical positions. The indirect access view argued for the literal meaning of a word to be always accessed first, even during figurative language processing. Therefore a prime word that is a property of the literal but not of the figurative meaning counts as a related prime. Literal priming therefore should elicit the same effects like semantic priming, i.e., a reduced or unchanged N400-amplitude difference. On the other side, the direct access view suggested that the literal meaning of the target word is not accessed in figurative processing. Therefore the literal prime can be equated with unrelated priming and should have no facilitating or even a hampering effect (more pronounced N400). The calculated difference wave plot (see Figure 4) compared the amplitude difference between the metaphoric and literal conditions without and with priming. They revealed that the literal prime word has no hampering effect (no enhanced amplitude) on the processing of figurative utterances. In contrast, the N400-amplitude was reduced. This observation is also supported by the processing patterns in the metonymy study, where masked priming resulted in the absence of a difference between the two conditions, indicating that the literal prime does not impede processing. The findings therefore point against accounts that argue for literal meaning to play no role or even having a negative influence on the processing of figurative utterances (e.g., Glucksberg, 2008; Sperber and Wilson, 2008). The data in turn support theories that integrate the literal meaning in the processing of metaphors and metonymies. This involves relatively strict accounts that propose a literal first step in their model (Grice, 1975; Searle, 1979), as well as accounts that argue for the lingering of literal meaning (Carston, 2010b). Our findings are also in line with theoretical approaches that argue for the literal meaning to have a role in blending, e.g., mapping processes or semantic/computational approaches (cf. Kintsch, 2000; Croft, 2002; Fauconnier and Turner, 2002; Coulson and Oakley, 2005; Wolff and Gentner, 2011).

The cross-modal priming technique adopted here for the first time therefore gave important insights into the role of literal meaning in figurative language processing. Namely it shows that during the lexical access phase (N400), independent of figurativity, the literal meaning is activated and therefore primes related to the literal meaning of the critical word facilitate lexical access (reduced N400). It is uncontroversial that the contextually relevant meaning is determined within a short period of time. Still, and crucially, the masked priming data revealed that literal meaning aspects are initially available regardless of whether they are contextually relevant or not. Interestingly, converging evidence comes from the literature on idioms: the literal meaning of the constituent words can be available until the end of idiom strings, and even after the idiomatic meaning has already been recognized (Cacciari, 2014). Theories about figurative language should thus include a phase in which the literal meaning of the critical word is accessed.

Beside the reduced N400-amplitude, literal priming in metaphors causes a delayed Late Positivity. Because priming in metonymies did not elicit a Late Positivity, we can exclude the possibility that literal priming *per se* results in a delayed Positivity. Based on the findings of Schumacher et al. (2012) who used the same paradigm, we argue that in sentential context primes influence processes beside lexical access that are already demanding when computed without priming. This would explain why we found a delayed Late Positivity in 1b (for metaphors) but not in 2b (for metonymies). The findings by Schumacher et al. (2012) then could be reinterpreted in terms of a delayed P325, which reflects the entire repetition of the auditory prime word by the visual target during lexical form processing (cf. Holcomb and Grainger, 2006). Additional studies, for instance with figurative prime words, are needed to shed further light on these findings.

In sum, our data indicate that literal meaning aspects are accessed during the processing of metaphor and metonymy. We further suggest that the electrophysiological differences observed between the ERP patterns in metaphor and metonymic processing call for a more refined typology of figurative processes. To this end, we discussed different types of metonymy (such as producer-for-product metonymy vs. reference transfer) and the possibility of different types of metaphor (nominal vs. verbal).

### Conflict of interest statement

The authors declare that the research was conducted in the absence of any commercial or financial relationships that could be construed as a potential conflict of interest.

## Footnotes

^1^We use literal meaning in the sense that it is the meaning of a word stored in the lexicon and which it has if taken for itself (“context-free”). This definition is based on Searle (1978, 1979), who stated that the components of sentences constitute their literal meaning. A single word and a sentence both could have more than one literal meaning, e.g., homonyms. Crucially it is important to differentiate between the literality of the whole utterance and the single words within as was e.g., pointed out by Gibbs (2002) and Recanati (1995). Literal meaning is often contrasted with figurative language in a sense that everything that is not literal is figurative. Beside this binary view, some approaches consider literal and figurative meaning as the endpoints on a scale on which the different phenomena (e.g., metaphor, metonymy, irony, idioms) can be arranged.

^2^A clear discrimination between literal and figurative (non-literal) sentences has been abandonned in the literature in favor of a continuum hypothesis between the figurative and literal pole (cf. e.g., Coulson and van Petten, 2002; Coulson, 2006; Rubio Fernández, 2007; Sperber and Wilson, 2008). Throughout this manuscript, we use the terms literal and figurative to discriminate between the two test conditions.

^3^Crucially, the fact that we found an N400-amplitude difference between the two conditions speaks for stimulus processing beyond the word level in both presentation modalities (without and with priming). If participants processed each word separately, we should not have found differences at hyena, neither in the condition without nor in the condition with priming.

^4^Although visual inspection suggests some later effect, statistical analyses with 50 ms windows from 350 to 900 ms revealed only a significant effect for the time-window between 400 and 450 ms (ROIs and midline) and for the time-window from 550 to 600 ms (ROIs only). Since no two adjacent time-windows elicited significant differences, we do not consider this difference reliable (cf. Gunter et al., 2000).

^5^Note that the suggestions by Nunberg (1995) did also include the stringent necessity of supporting context for the processing of reference transfer, but this was refuted by Schumacher (2014).

^6^Evidence for referent deletion comes from pronoun tests that indicate that “hyena” is no longer accessible: My boss is a hyena. #It is aggressive.
