# Supplementary material for: The role of literal meaning in figurative language comprehension: evidence from masked priming ERP
Source: Front Hum Neurosci. 2014 Aug 4;8:583. doi: 10.3389/fnhum.2014.00583 (PMC4120764; doi:10.3389/fnhum.2014.00583)
Supplement: Supplementary file 1 [file DataSheet1.PDF]

Supplementary Material

The role of literal meaning in figurative language comprehension: Evidence from masked priming ERP

Hanna Weiland[1] [1,]*, Valentina Bambini[2] [2], Petra B. Schumacher[3] [1]

[1]Department of English and Linguistics, Johannes Gutenberg University Mainz, Mainz, Germany

[2]Center for Neurocognition and Theoretical Syntax, Institute for Advanced Study, IUSS Pavia, Italy

***Correspondence:** Hanna Weiland, Department of English and Linguistics, Johannes Gutenberg University Mainz, Jakob-Welder-Weg. 18, 55099 Mainz, Germany. weiland@uni-mainz.de

1. Supplementary Tables

Supplementary Table 1. Stimuli of Experiment 1. Material of Experiment 1a & b, including the respective target word for the literal and the figurative condition, the critical word (vehicle) and the prime word, as well as the corresponding averaged property coherence value (ranging from *no coherence* (1) to *strong coherence* (6) and standard deviation (SD).

**item**	**target lit. cond.**	**target fig. cond.**	**critical word**	**property/prime**	**coherence**	**SD**
1	Insekten	Menschen	Ameisen	krabbelnd	5,58	0,78
1	*insects*	*humans*	*Ants*	*crawling*		
2	Pflanzen	Melodien	Baldrian	natürlich	4,5	1,38
2	*plants*	*melodies*	*Valerian*	*natural*		
3	Naturgebilde	Kuchen	Berge	felsig	5,63	0,77
3	*natural formations*	*cake*	*Mountains*	*rocky/cliffy*		
4	Insekten	Sekretärinnen	Bienen	gestreift	5,67	0,56
4	*insects*	*secretaries*	*Bees*	*striped*		
5	Säugetiere	Kinder	Böcke	gehörnt	5,63	1,06
5	*mammals*	*children*	*Bucks*	*cornute*		
6	Apparate	Köpfe	Computer	digital	5,46	0,78
6	*instruments*	*heads*	*Computer*	*digital*		
7	Pflanzenteile	Erinnerungen	Dornen	pieksig	6	0
7	*parts of plants*	*memories*	*Thorns*	*piercing*		
8	Rauschmittel	Lieder	Drogen	illegal	5,42	0,72
8	*intoxicants*	*songs*	*Drugs*	*illegal*		

item	target lit. cond.	target fig. cond.	critical word	property/prime	coherence	SD
9	Bäume	Ehen	Eichen	wurzelig	5,24	1,26
9	*trees*	*marriages*	*oak trees*	*rooty*		
10	Säugetiere	Kletterer	Eichhörnchen	putzig	5,59	0,67
10	*mammals*	*climbers*	*squirrels*	*cute*		
11	Lebensgemeinschaften	Firmen	Familien	verwandt	5,5	0,96
11	*communities*	*companies*	*families*	*related*		
12	Öffnungen	Augen	Fenster	kippbar	5,86	0,45
12	*openings*	*eyes*	*window*	*titlable*		
13	Pyrotechniken	Konzerte	Feuerwerke	farbenfroh	5,33	1,05
13	*pyrotechnics*	*concerts*	*fireworks*	*colourful*		
14	Meerestiere	Menschen	Fische	schuppig	5,83	0,48
14	*marine animals*	*humans*	*fishes*	*scaly*		
15	Säugetiere	Diebe	Füchse	rötlich	5,67	0,64
15	*mammals*	*thieves*	*foxes*	*reddish*		
16	Bauwerke	Theorien	Gebäude	renoviert	5,05	1,36
16	*architectures/buildings*	*theories*	*buildings*	*renovated*		
17	Bauwerke	Jobs	Gefängnisse	bewacht	5,71	0,62
17	*architectures/buildings*	*joby*	*jails*	*watched*		
18	Raubtiere	Lobbyisten	Hyänen	fellig	5,14	0,99
18	*predators*	*lobbyists*	*hyaenas*	*skinned*		
19	Behälter	Ehen	Käfige	vergittert	5,75	0,44
19	*containers*	*marriages*	*cages*	*barred*		
20	Behälter	Erinnerungen	Konserven	luftdicht	5,82	0,5
20	*containers*	*memories*	*cans*	*air tight*		
21	Naturgebilde	Körper	Landschaften	grün	5	1,02
21	*natural formations*	*bodys*	*landscapes*	*green*		
22	Bücher	Professoren	Lexika	gebunden	5,36	0,95
22	*books*	*professors*	*lexicons*	*bound*		
23	Raubtiere	Soldaten	Löwen	mähnig	5,63	1,1
23	*predators*	*soldiers*	*lions*	*maned*		
24	Steine	Politiker	Magnete	zweipolig	5,92	0,28
24	*stones*	*politicians*	*magnets*	*bipolar*		

item	target lit. cond.	target fig. cond.	critical word	property/prime	coherence	SD
25	Bauwerke	Konservativen	Mauern	massiv	5,5	1,1
25	*architectures/buildings*	*cans*	*walls*	*massive*		
26	Werkzeuge	Worte	Messerklingen	gewetzt	5,68	0,65
26	*instruments*	*words*	*knife blades*	*sharpened*		
27	Vögel	Tänzerinnen	Pfauen	gefiedert	5,38	1,06
27	*birds*	*dancers*	*peacocks*	*feathered*		
28	Waffen	Autos	Pfeile	spitz	5,87	0,34
28	*weapons*	*cars*	*arrows*	*spiky*		
29	Materialien	Wangen	Porzellan	glasiert	5,05	1,53
29	*materials*	*cheeks*	*porcelain*	*glazed*		
30	Spiele	Verbrechen	Puzzles	flach	4,46	0,93
30	*games*	*crimes*	*puzzles*	*flat*		
31	Blumen	Mädchen	Rosen	duftend	5,54	0,78
31	*flowers*	*girls*	*roses*	*fragrant*		
32	Spielsteine	Soldaten	Schachfiguren	hölzern	4,38	1,13
32	*gaming pieces*	*soldiers*	*pawns*	*wooden*		
33	Arzneimittel	Schulstunden	Schlafpillen	rund	4,57	1,12
33	*pharmaceuticals*	*lessons*	*sleeping pills*	*round*		
34	Naturgebilde	Falten	Schluchten	steil	5,64	0,95
34	*natural formations*	*wrinkles*	*gorges*	*steep*		
35	Insekten	Tänzerinnen	Schmetterlinge	fliegend	5,81	0,39
35	*insects*	*dancers*	*butterflies*	*flying*		
36	Naturphänomene	Töchter	Sonnenscheine	hell	5,83	0,38
36	*natural phenomena*	*daughters*	*sunshines*	*light*		
37	Vögel	Liebespaare	Tauben	fliegend	5,88	0,34
37	*birds*	*lovers*	*pigeons*	*flying*		
38	Fantasiewesen	Bankiers	Vampire	gruselig	5,32	0,72
38	*fanatsy character*	*bankers*	*vampires*	*creepy*		
39	Naturphänomene	Proteste	Wellen	blau	4,75	1,15
39	*natural phenomena*	*protests*	*waves*	*blue*		
40	Personen	Bäume	Zeugen	gesucht	4,67	1,43
40	*people*	*trees*	*witnesses*	*wanted /searched*		

Supplementary Table 2. Stimuli of Experiment 2. Material of Experiment 2a & b, including the critical word and the prime word, as well as the corresponding averaged property coherence value (ranging from *no coherence* (1) to *strong coherence* (6) and standard deviation (SD).

item	critical word	property/prime	coherence	SD
1	Armani	gebräunt	3,04	1,74
1		*bronzed*		
2	Beethoven	taub	5,29	0,95
2		*deaf*		
3	Böll	talentiert	3,75	1,33
3		*talented*		
4	Brahms	bärtig	3,65	1,72
4		*bearded*		
5	Brecht	menschlich	4,27	1,8
5		*human*		
6	Büchner	verlobt	2,14	1,35
6		*engaged*		
7	Chopin	begabt	4,57	1,21
7		*gifted*		
8	Dali	verwitwet	2,3	1,4
8		*widowed*		
9	DaVinci	vollbärtig	3,83	1,4
9		*full bearded*		
10	Dior	menschlich	2,29	1,46
10		*human*		
11	Döblin	studiert	3,57	1,86
11		*studied*		
12	Goethe	studiert	4,5	1,44
12		*studied*		
13	Grass	geschieden	2,14	1,59
13		*divorced*		
14	Gucci	bartlos	2,57	1,72
14		*beardless*		

**item**	**critical word**	**property/prime**	**coherence**	**SD**
15	Händel	erblindet	2,92	1,47
15		*go blind*		
16	Haydn	kränkelnd	2,35	1,53
16		*ailing*		
17	Hesse	menschlich	4,68	1,76
17		*human*		
18	Heine	studiert	3,63	1,81
18		*studied*		
19	Joop	arrogant	4,29	1,49
19		*arrogant*		
20	Kafka	verlobt	2,6	1,14
20		*engaged*		
21	Kandinsky	menschlich	3,91	2,02
21		*human*		
22	Kästner	studiert	3,58	1,35
22		*studied*		
23	Kleist	verarmt	2,82	1,91
23		*destitute*		
24	Lagerfeld	arrogant	5,09	1,43
24		*arrogant*		
25	Michelangelo	bärtig	3,38	1,41
25		*bearded*		
26	Monet	erblindet	3,18	1,62
26		*amaurotic*		
27	Mozart	menschlich	4,86	1,58
27		*human*		
28	Picasso	fleißig	3,79	1,84
28		*diligently*		
29	Rembrandt	verwitwet	2,74	1,79
29		*widowed*		
30	Rubens	menschlich	3,58	1,64
30		*human*		

**item**	**critical word**	**property/prime**	**coherence**	**SD**
31	Schiller	menschlich	5,18	1,3
31		*human*		
32	Schubert	erkrankt	3,09	1,82
32		*diseased*		
33	Strauss	menschlich	3,77	2,05
33		*human*		
34	Süskind	begabt	3,81	1,81
34		*gifted*		
35	Tschaikowsky	studiert	3,63	1,17
35		*studied*		
36	Van Gogh	begabt	4,52	1,86
36		*gifted*		
37	Verdi	menschlich	4	1,95
37		*human*		
38	Versace	schwul	2,79	1,96
38		*gay*		
39	Vivaldi	kränkelnd	2,86	1,42
39		*ailing*		
40	Wagner	menschlich	5,05	1,43
40		*human*		
